# Biochemical parameters and oxidative stress markers in Tunisian patients with periodontal disease

**DOI:** 10.1186/s12903-019-0912-4

**Published:** 2019-10-22

**Authors:** Ahmed Gharbi, Ali Hamila, Adel Bouguezzi, Azza Dandana, Salima Ferchichi, Fatiha Chandad, Leila Guezguez, Abdelhedi Miled

**Affiliations:** 1 Biochemistry Laboratory CHU Farhat Hached, Sousse, Tunisia; 2 Biological and Clinical Dento-facial Approach Laboratory LR12ES10, Monastir, Tunisia; 3 Laboratory of Physiology, Faculty of Medicine Sousse, Sousse, Tunisia; 4 Department of Oral Medicine and Surgery, School of Dental Medicine, University of Monastir, Sousse, Tunisia; 5 Oral Ecology Research Group, Faculty of Dental Medicine, Laval University, Sousse, Tunisia; 6 Department of Periodontology, School of Dental Medicine, University of Monastir, Sousse, Tunisia

**Keywords:** Periodontal disease, Oxidative stress, GR, TOAC, Antioxidant, Periodontitis

## Abstract

**Background:**

Oxidative stress is involved in many diseases including diabetes and cancer. Numbers of studies have suggested its involvement in the pathogenesis of periodontal diseases. The aim of this study was to evaluate the levels of biochemical parameters and oxidative stress markers in plasma of healthy and chronic periodontitis patients.

**Methods:**

One hundred thirty subjects were divided into two groups; patients (mean age = 42 ± 13.6 y.o) and control (mean age = 44.8 ± 12.6 y.o). Patients and healthy subjects were free from any infection, coronary or heart disease, diabetes or liver failure. Total cholesterol, LDLc, HDLc, Triglycerides (TG), creatinine, uric acid (UA), glucose and urea levels as well as the activities of enzymatic antioxidants such as catalase, glutathione reductase (GR) and total antioxidant capacity (TAOC), were measured in plasma samples using colorimetric assays. Statistical differences between groups were determined by Student’s t-test and *p* ≤ 0.05 was considered as significant.

**Results:**

Periodontitis patients exhibited significant decrease in the activities of catalase, TAOC, GR and TG, cholesterol, LDLc, glucose, HDLc, uric acid levels in plasma samples in comparison with healthy subjects. However, no statistically significant differences in the levels of creatinine and urea were observed between the two groups.

**Conclusion:**

The reduction of plasma antioxidant activities (Catalase, TAOC, GR) may have a role in the pathogenesis of periodontal diseases. Our findings suggest a decrease in the host capacity to control the damage caused by oxidative stress. Therefore, therapeutic strategies, aiming at modulating the oxidative stress could be considered as potential tools for the prevention or treatment of periodontal diseases and their potential systemic effects on the general health.

## Introduction

Periodontitis is known as an inflammatory disease that affects oral health. It’s the leading cause of tooth loss, secondary to periodontal pocket formation and alveolar bone resorption. In high income countries, the disease affects 10–15% of adults [1, 2]. The pathogenesis stems from a combination of factors, involving periodontal bacteria, inflammatory immune responses, patient behavioral (life style) and concomitant medical conditions. More recently this pathology has been linked to several metabolic diseases, including diabetes and cardio-vascular diseases. The association between aging and periodontitis is not conclusively proved [1, 3].

Recently, it has been suggested that the increase of inflammation accompanying periodontitis is considered as a risk factor for certain systemic diseases such as diabetes type 2 [3, 4], cardiovascular and cerebrovascular diseases [5, 6]. On the Other hand it has been demonstrated that oxidative stress triggers the chronic state of periodontitis as evidenced by an increased levels of biomarkers for tissue damage [7, 8].

Chronic and aggressive periodontitis are characterized by an increased production of reactive oxygen species (ROS), which may be attributed to the functional activation of polymorphonuclear leucocytes (PMNLs) [6, 7]. To date, no explanation has been provided regarding the involvement of ROS production in the progression of periodontal diseases, but its impact cannot be considered in isolation, as various antioxidant species are involved in the defense mechanisms against excess ROS activity and maintain a delicate equilibrium within the host tissues [9]. Many studies have reported that these molecules are capable of inducing the destruction of periodontal tissues and are associated with osteoclastic bone resorption, as well [8].

Its well known that inflammation is strongly associated to increased oxidative stress components in periodontal disease. Thus, an uncontrolled inflammatory response in periodontitis can serve as an intermediate variable between them [10].

The aim of this study was to evaluate the levels of biochemical parameters and oxidative stress markers in plasma of healthy and chronic periodontitis patients in a Tunisian population.

## Patients and methods

### Study groups

The current study included a total of one hundred thirty subjects (130), comprising 80 periodontitis patients (PG, 47 females and 33 males, aged from 20 to 60 years), and 50 periodontal healthy controls (CG, 25 females and 25 males, aged from 20 to 60 years). The recruited participants were selected among individuals who were referred to the Faculty of dental medicine (Monastir University, Tunisia) for periodontal problems or routine periodontal controls from October 2013 to April 2014. The Subjects admitted to the dental clinic for routine periodontal follow-up and the individuals without any periodontal problems were included in the control group.

All the patients involved in the study had no history of systemic condition, alcohol and tobacco consumption or any drug intake, as previously reported [11]. The participants were informed of the study, and they gave their informed consent.

### Clinical measurements

Clinical and radiological diagnosis for periodontal diseases were performed according to the criteria of the American Academy of Periodontology [12, 13]. The periodontal status of all the individuals was determined by measuring the probing pocket depth (PPD) using a periodontal probe (PCP-UNC15, Hu-friedy), the gingival bleeding index (GBI), and the plaque index (PI). The diagnosis of chronic periodontitis was made based on the following criteria: clinical attachment loss (CAL), 3 to 4 mm, radiographic periodontal bone loss (coronal third), 15 to 33% and a PPD of ≤5 mm.

### Collection of blood samples

Blood samples were collected 48 h after the clinical measurements, in the morning and after an overnight fasting.

Venous blood was collected in plain tubes (for serum). All tubes were maintained at 4 °C for 30 min before centrifugation at 1500 xg for 10 min. Serum samples were collected, aliquoted into cryogenic vials and stored in − 20 °C.

### Measurement of biochemical variables

The Levels of Cholesterol (TC), triglyceride (TG), high-density lipoprotein cholesterol (HDLc), low-density lipoprotein cholesterol (LDLc), creatinine, uric acid, glucose and serum urea were measured using standardized enzymatic methods (Randox-Antrim, UK).

### Determination of the oxidative state markers

TAOC levels of serum samples were measured using commercially available human-specific enzyme-linked immunosorbent assays (ELISA), following the instructions of the manufacturer (Cayman Chemical, Ann Arbor, MI, USA). The concentration values in serum were expressed as milli molar Trolox equivalent for TAOC.

Glutathione reductase (GR) activity was determined by measuring the rate of NADPH oxidation using a GR assay kit (Cayman Chemical, Ann Arbor, MI, USA).

The catalase (CAT) activity was determined spectrophotometrically as the rate of substrate decomposition per unit time [14].

### Statistical analysis

All entered data were analysed using SPSS software (Statistical Package for the Social Sciences version 15 USA). The kolmogorov-Smirnov normality test was used to assess data distribution. Variables with a normal distribution were expressed as means±SD or standard error 95% Confidence Interval and then compared using the Student’s t-test for independent samples. Qualitative variables were expressed as frequencies and percentages. The Chi2 test was used to compare qualitative data. The level of significance was set at *p* ≤ 0.05.

## Results

### Clinical findings

The mean values of clinical parameters are reported in Table 1. Mean values of PPD, GBI and PI were significantly higher in the PG compared to the CG, However, No significant difference was observed for both groups according to the gender (*p ≤ 0.05*).

**Table 1:** Comparison of clinical periodontal parameters among the groups

Groups Parameter	Patients *n* = 80	Controls *n* = 50	*P*	95%CI
	Median			
PPD (mm)	5.3 (2.5 to 7.5)	1 (0.5 to 2)	0.05	4.99–0.70
GBI	1.96 (0 to 3)	0.75 (0 to 1)	0.05	2.38–0.67
PI	64.4 (10 to 100)	35 (10 to 100)	0.05	65.81–34.71

Patients *n* = 80; Controls *n* = 50. Normally distributed data are expressed as means±SD, and non normally distributed data are expressed as median (IQR [interquartile range]). *PPD* Probing pocket depth, *GBI* Gingival bleeding index, *PI* Plaque index. The difference is significant compared with the control group (*P* ≤ 0.05)

### Biochemical parameters

The mean concentration of plasma TG and HDlc, CT, LDLc, Levels from the PG and CG are given in Table 2. The TG (*p* ≤ 0.05), LDLc (*p* < 0.05) and TC (*p* < 0.05) concentrations were significantly higher in the PG compared to the CG. The plasma HDLc concentration was statistically decreasing in the PG compared with the CG (*p* < 0.05).

**Table 2:** Comparison of plasma concentrations of CT, LDLc, TG and HDLc levels among the groups

Groups Value	Patients	Controls	*P*	95%CI
	Values			
Plasma TC (mmol/l)	4.75 ± 1.10	4.2 ± 0.86	0.02	4.40–4.73
Plasma LDLc (mmol/l)	2.3 ± 0.72	2.08 ± 0.86	0.05	2.30–2.57
Plasma TG (mmol/l)	1.4 ± 0.91	1.1 ± 0.56	0.05	1.19–1.42
Plasma HDLc (mmol/l)	1.3 ± 0.41	1.41 ± 0.43	0.03	1.14–1.28

Patients, *n* = 80; Controls, *n* = 50. Normally distributed data are expressed as means±SD, and non normally distributed data are expressed as median (IQR [interquartile range]). *CT* Cholesterol, *LDLc* Low-density lipoprotein cholesterol, *TG* Triglycerides, *HDLc* High-density lipoprotein cholesterol. The difference is significant compared with the control group (*P* ≤ 0.05)

The mean concentration of plasma creatinine, uric acid, glucose and urea levels from the patients and control groups are given in Table 3. The plasma glucose and uric acid levels were statistically higher among the PG compared with the CG (*p* ≤ 0.05).

**Table 3:** Comparison of creatinine, uric acid, glucose and urea in plasma among the groups

Groups Value	Patients	Controls	*P* value	95%CI
	Values			
Plasma creatinine (μmol/l)	64.93 ± 15.25	66.62 ± 10.35	0.13	63.62–67.97
Plasma Uric Acid (mmol/l)	310.37 ± 53.86	245.82 ± 66	0.05	275.35–295.66
Plasma Glucose (mmol/l)	4.8 ± 1	3.9 ± 0.80	0.05	4.41–4.67
Plasma Urea (μmol/l)	4.60 ± 1.36	5.06 ± 0.56	0.31	4.40–4.89

Patients, *n* = 80; Controls, *n* = 50. Normally distributed data are expressed as means±SD, and non normally distributed data are expressed as median (IQR [interquartile range]).The difference is significant compared with the control group (*P* ≤ 0.05)

There was no statistically significant difference for the plasma creatinine and urea in the PG compared with the CG (*p* > 0.05).

The plasma mean levels of enzymatic antioxidants (catalase, GR and TAOC) in plasma among the groups are given in Table 4. The index values showed strong statistically significant difference among the groups (*p* < 0.05). The values were significantly higher in the CG than in the PG.

**Table 4:** Comparison of the activities of enzymatic antioxidants (Catalase, GR and TAOC) in plasma among the groups

Groups Value	Patients	Controls	*P* value	95%CI
	Values			
Plasma catalase (ku/l)	1.95 ± 0.40	13.38 ± 10	0.001	5.49–8.46
Plasma GR (nmol/min/ml)	28.55 ± 10.79	48.72 ± 8.02	0.001	33.91–38.72
Plasma TAOC (mM trolox equivalent)	2427 ± 520.31	4192 ± 465	0.002	2933.42–3279.01

Patients, *n* = 80; Controls, *n* = 50. Normally distributed data are expressed as means±SD, and non normally distributed data are expressed as median (IQR [interquartile range]). *GR* Glutathione reductase, *TAOC* Total antioxidant capacity. The difference is significant compared with the control group (*P* ≤ 0.05)

## Discussion

Periodontitis is an inflammatory disease of infectious nature affecting the tooth supporting tissues. The presence of periodontopathogenic bacteria in the gingival crevice is the primary etiological factor responsible for triggering periodontitis. The immune response of the host to the aggression by these periodontopathogenic agents determines the course of the disease to the tissue destruction or recovery.

In our study, biochemical parameters showed an increase in cholesterol, LDLc, glucose and triglyceride and a decrease for HDLc. However, no significant difference was found for creatinine and urea.

### Study of the lipid profile

The mean values of total plasma cholesterol (TC), LDLc were significantly higher in patients while HDLc was significantly lower compared to controls.

These results corroborate those reported by Dhotre et al. [15] and Lösche et al. [16].

Jaramillo et al. [17] found that untreated periodontal diseases were associated with altered lipid markers related to cardiovascular disorders.

However, the study conducted by Gita et al. in 2012 showed no association between periodontal diseases and total cholesterol, LDLc, HDLc and triglycerides levels [18].

The disturbance of the lipidic metabolism could be explained by the inhibition of the lipase lipoprotein secondary to the release of pro-inflammatory cytokines, such as IL-1β and TNF-α during periodontal diseases. Furthermore, the increase of the cardiovascular disease risk might be due to the impairment of the HDLc antiatherogenic properties.

### Urea and creatinine

Our results showed, as recorded by Brotto et al. [19], no significant difference in the plasma urea and creatinine levels between the two groups.

Several studies have shown a positive relationship between periodontitis and abnormally high serum creatinine associated with renal dysfunction. For instance, in 2005, Kshirsagar et al. [20] demonstrated a correlation between periodontitis and kidney failure and the results showed a significant increase in the levels of creatinine in patients compared to controls.

### Glucose

In our study we have shown a significant increase in blood glucose levels in patients compared to controls.

These results are consistent with those described by Lösche et al. [16]. These observations may indicate that these patients were in a pre-diabetic condition and had uncontrolled blood glucose. In fact, poor blood sugar control is known as an established risk factor for periodontitis.

### Study of oxidative stress markers

ROS are known to be involved in periodontal diseases, and their effect will be modulated in vivo by the antioxidant defense system [21].

### Catalase

Our results showed a statistically significant decrease in erythrocyte catalase activity. These results are in line with those recorded by Thomas et al. [22]. This could explain the decrease in the catalase for patients with periodontal diseases at an advanced stage.

Conversely, a study conducted by Panjamurthy et al. [3] showed that the level of catalase and other enzymatic antioxidants (SOD) were elevated in patients compared to controls.

In this regard, it is well known that the antioxidant reactions found in different pathologies depend on the severity or spread of the disease [23, 24].

### Uric acid

Uric acid is an antioxidant molecule with powerful reducing free radicals [25]. In our study, a significant increase in the uric acid level was found in patients compared to controls. These results corroborate with those found by Ziebolz et al. [26] and Banu Sand et al. [27], who considered that uric acid could be used as a readily detectable mediator of inflammation in periodontitis.

However Brotto et al. [19] showed a non-significant increase of uric acid in patients compared to controls.

### Glutathione reductase (GR)

Reduced glutathione is an antioxidant marker, with multiple biological functions. It prevents oxidative stress, removes hydroperoxides, detoxifies and stabilizes biological membranes. Therefore, our results are in accordance with those of Chapple et al. [9] who reported that patients having periodontal disease show a lower concentration of glutathione in their serum and gingival crevicular fluid.

### TOAC

The results of our study for serum TAOC levels are in agreement with the observations of Chapple et al. [2] and Brock et al. [21]. The authors showed that plasma TAOC levels were significantly reduced in patients with chronic periodontitis (*P* ≤ 0.001) and gingivitis (P ≤ 0.001) when compared with age and sex matched healthy controls. The destruction can be explained by an imbalance between the production of the antioxidant and the ROS. The increased of ROS production is a result of the inflammatory infiltrate of PMNs as a host response against microbial invasion. The infiltration of PMN in numbers is likely to result in an increase in ROS levels causing oxidative stress. Thomas et al. [22] reported that the increased gingival crevicular fluid lipid peroxidation levels reflected the increased ROS damage to the periodontal tissues and lead to periodontal inflammation.

Hence, oxidative stress lies at the heart of the periodontal tissue. It results from host–microbial interactions. It can be either direct resulting from excess ROS activity/antioxidant deficiency or indirect arising from the activation of redox-sensitive transcription factors and the creation of a pro-inflammatory state.

Taking these data as well as our results into consideration, we hypothesize that periodontal disease may be a potential risk factor for the severity, progression and even the onset of cardiovascular diseases. This is due to a reduced antioxidant capacity and an increased oxidative stress and atherogenic lipoproteins [16].

## Conclusion

- Plasma antioxidant activities (catalase, TAOC, GR) are decreased in the periodontally compromised group compared with the control group,
- Our findings suggest a decrease in the host capacity to control the damage caused by oxidative stress,
- Further studies are needed to better evaluate the role of reactive oxygen species in the pathogenesis of chronic periodontitis and their potential systemic effects on the general health.

## Data Availability

All data generated or analyzed during this study are included in this published article are available from the corresponding author on reasonable request.

## Abbreviations

CAL: Clinical attachment loss
CAT: Catalase
GBI: Gingival bleeding index
GR: Glutathione reductase
HDLc: High-density lipoprotein cholesterol
LDLc: Low-density lipoprotein cholesterol
MDA: Malondialdehyde
PI: Plaque index
PMNLs: Polymorphonuclear leucocytes
PPD: Periodontal probing depth
ROS: Reactive oxygen species
SOD: Superoxide dismutase
TAOC: Total antioxidant capacity
TBA: Thiobarbituric acid
TC: The Levels of Cholesterol
TG: Triglycerides
UA: Uric acid
